# Enhancement of photocatalytic efficiency of copper oxide/zinc oxide-montmorillonite photocatalyst under visible light irradiation

**DOI:** 10.1080/14686996.2025.2469484

**Published:** 2025-02-21

**Authors:** Chomponoot Suppaso, Nipaporn Pongkan, Sonchai Intachai, Wachiraya Rattanawongsa, Areebhorn Baoulan, Yusuke Yamauchi, Yusuke Asakura, Nithima Khaorapapong

**Affiliations:** a Department of Chemistry and Center of Excellence for Innovation in Chemistry, Faculty of Science, Materials Chemistry Research Center, Khon Kaen, Thailand; b Department of Chemistry, Faculty of Science and Digital Innovation, Thaksin University, Phatthalung, Thailand; c Department of Chemistry, Faculty of Engineering, Rajamangala University of Technology Isan, Khon Kaen, Thailand; d Department of Materials Process Engineering, Graduate School of Engineering, Nagoya University, Nagoya, Aichi, Japan; e Australian Institute for Bioengineering and Nanotechnology (AIBN) & School of Chemical Engineering, The University of Queensland (UQ), Queensland, Australia

**Keywords:** Copper oxide, zinc oxide, montmorillonite, photocatalysts, methylene blue

## Abstract

The formation of copper oxide and zinc oxide mixture in montmorillonite was conducted by the reaction of an aqueous dispersion of Cu^2+^/Zn^2+^ exchanged montmorillonite and an aqueous solution of sodium hydroxide under hydrothermal treatment. The resulting product was characterized by X-ray diffraction, scanning and transmittance electron microscopies, as well as UV-visible and photoluminescence spectroscopies. The diffuse reflectance absorption spectra showed the absorption onsets due to copper oxide (885 nm) and zinc oxide (310 and 580 nm) in the product. The adsorption of methylene blue was fitted well by the Langmuir model with the maximum adsorption capacity of 454 mg⋅g^−1^. The thermodynamic studies revealed that the process is exothermic and spontaneous. The photocatalytic activity of the hybrid was assessed by the degradation of methylene blue in aqueous solution under visible light irradiation. The most active species in the photocatalytic process was hydroxyl radicals. The regenerated copper oxide/zinc oxide-montmorillonite was reused up to 5 cycles, the photodegradation efficiency dropped only 5% (from 94% to 89%), supporting the good stability of the photocatalyst. The result was in agreement with the advantages of the nanocomposite heterostructure and the unique nature of montmorillonite.

## IMPACT STATEMENT

CuO/ZnO/montmorillonite hybrid prepared through intercalation chemistry exhibited efficient charge transfer of photoexcited carriers under visible light irradiation, resulting in enhanced MB degradation. The efficiency decreased by only 5% after 5 cycles.

## GRAPHICAL ABSTRACT

**Figure:**
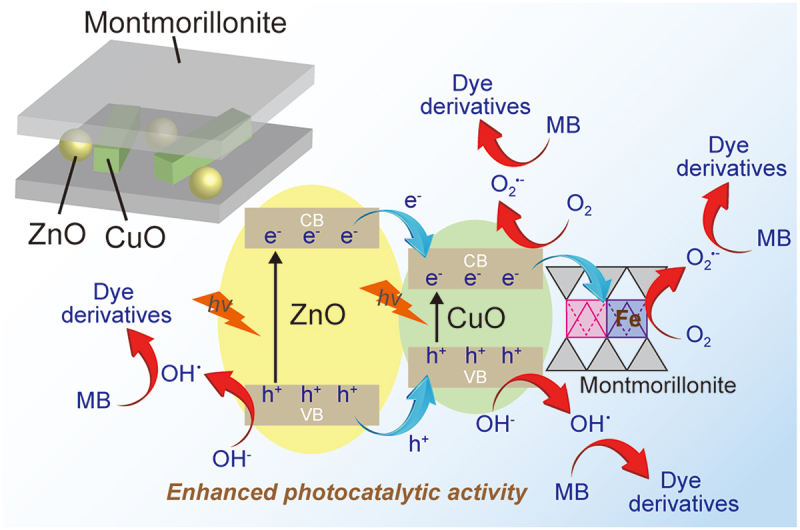

## 1. Introduction

Metal oxide photocatalysts, especially zinc oxide (ZnO) and titanium dioxide (TiO_2_), have received much interest over the past decades due to promising allocations in environmental remediation, including water purification. The major limitation of these oxides lies in their large band gaps (3.2–3.4 eV), which restrict their excitation to UV light, rendering them inefficient or inactive ones under visible and solar light exposures (~390–800 nm). To overcome this significant drawback, extensive research has been conducted to expand the optical response of those metal oxides from UV to visible region by doping with metal or nonmetal ions into their lattices [1,2], combining them with another visible light active metal oxide [3], and/or immobilizing them on solid supports [2,4,5]. Copper oxide (CuO), with its narrow band gap of 1.2 eV, exhibits photoefficiency for visible light. Composite of metal oxides created two distinct energy levels that play a crucial role in facilitating charge separation and transportation in the heterostructures under irradiation, thereby enhancing photocatalytic activity [5–8].

Montmorillonite, a type of 2:1 layered phyllosilicates has obtained much attention owing to its swelling behavior, cation exchange property, and large surface area, making it highly efficient as an adsorbent and solid support [9]. Several studies have successfully demonstrated the modification of optical and/or catalytic properties of metal oxides (ZnO, CuO, Fe_2_O_3,_ or Co_3_O_4_) through the spatial distribution within montmorillonite [10–15]. In photocatalytic processes, a large surface area is essential for enhancing the rate of redox reaction, which accelerates the photocatalytic degradation process. The incorporation of CuO and ZnO within montmorillonite presents a promising and challenging approach for enhancing surface area and charge transfer across heterojunctions, ultimately improving photocatalytic efficiency. In spite of CuO and/or ZnO being primarily deposited on the outer surface of montmorillonite, they have proven to be effective photocatalysts for the degradation of methylene blue [14,15]. Additionally, our previous work demonstrated that ZnO incorporated within montmorillonite can effectively decompose methylene blue, even under acidic conditions [15]. Utilization of a supporting material such as montmorillonite for mixed metal oxides is crucial to mitigating their limitations and enhancing their overall performance.

In this work, a metal oxide mixture (CuO and ZnO) embedded in montmorillonite was synthesized using hydrothermal method, and its photocatalytic performance for the removal of methylene blue from aqueous solution was investigated. A significant challenge for metal oxide photocatalysts is their tendency to aggregate, as well as their limited response to visible light. The physicochemical properties of CuO and ZnO, including photocatalytic activity, aggregation, and surface area can be significantly enhanced by incorporating these oxides onto or within a solid support [10–12,16]. The decoration of CuO/ZnO on materials such as reduced graphene oxide, graphitic carbon nitride, and perlite has been shown to improve photodegradation activity under visible light irradiation [17–19]. However, the synthesis of mixed CuO/ZnO-modified clays has not yet been extensively reported. Herein, the integration of CuO and montmorillonite with ZnO may offer the potential to shift the active region of ZnO from UV to visible light, increase the specific surface area, and reduce the aggregation of semiconductor particles, thereby enhancing the photodegradation efficiency. In addition, the removal of methylene blue dye from an aqueous solution was achieved through dark adsorption followed by visible light-induced photodegradation using CuO/ZnO-montmorillonite.

## 2. Experimental

### 2.1. Materials

Natural montmorillonite (Kunipia F, Kunimine Industries, the reference clay sample of the Clay Science Society of Japan; JCSS-3101, Japan) was used as the host material, with a cation exchange capacity (CEC) of 1.20 meq·g^−1^. Copper chloride dihydrate (CuCl_2_·2H_2_O), zinc chloride (ZnCl_2_), and sodium hydroxide (NaOH) were sourced from Carlo Erba Reagent, Italy, while methylene blue (MB, C_16_H_18_ClN_3_S⋅H_2_O) was obtained from Riedel-de Haën, Germany. All chemicals are reagent grade and were directly used without any purification.

### 2.2. Hydrothermal synthesis of CuO/ZnO, CuO and ZnO photocatalysts

CuO/ZnO mixture was synthesized using a hydrothermal method. An aqueous solution containing metal chlorides (CuCl_2_⋅2H_2_O and ZnCl_2_) and NaOH with a molar ratio of Cu^2+^:Zn^2+^:OH^–^ = 1:1:4, was stirred magnetically at room temperature for 5 min. The resulting slurry was transferred to a Teflon-lined autoclave and subjected to hydrothermal treatment at 150°C for 24 h. The CuO/ZnO precipitate was collected by centrifugation, thoroughly washed with deionized water for several times to remove excess ions, and dried at 60°C. Bare CuO or ZnO was synthesized using the same procedure, but without the addition of ZnCl_2_ or CuCl_2_⋅2H_2_O, respectively.

### 2.3. Formation of mixed CuO/ZnO, CuO, and ZnO in montmorillonite

The incorporation of mixed CuO/ZnO into montmorillonite was performed using a hydrothermal reaction. The procedures were divided into two steps. Firstly, the ion exchange of Na^+^ in montmorillonite was carried out with divalent Cu^2+^ and Zn^2+^ cations. Aqueous solutions of metal salts (CuCl_2_·2H_2_O and ZnCl_2_) and a Na^+^-montmorillonite dispersion were mixed and magnetically stirred at room temperature for 24 h. The loading of Cu^2+^ and Zn^2+^ ions was adjusted to 50% CEC of montmorillonite. The resulting Cu^2+^/Zn^2+^-montmorillonite solid was collected by centrifugation, washed several times with deionized water, and dried at 60°C. In the next step, NaOH solution was added to the colloidal Cu^2+^/Zn^2+^-montmorillonite, followed by stirring for 5 min, with the molar ratio of Cu^2+^:Zn^2+^:OH^–^ = 1:1:4. The reaction mixture was then hydrothermally treated at 150°C for 24 h. The resulting precipitate was collected by centrifugation, washed thoroughly with deionized water several times, and dried at 60°C. The product was labelled as CuO/ZnO-montmorillonite. CuO-montmorillonite and ZnO-montmorillonite were also prepared using the same procedure, but without the addition of ZnCl_2_ or CuCl_2_·2H_2_O, respectively.

### 2.4. Adsorption study

The adsorption capacity, isotherm, thermodynamic, and kinetic behavior of the adsorbent toward methylene blue (MB) were investigated, with the reaction pH controlled at 7 ± 0.5. In typical experiment, 6 mg of the solid sample was added into 300 mL of an MB aqueous solution (30 ppm) and magnetically stirred in the dark to prevent photolysis of MB. The residual dye in the solution was measured using an Agilent HP 8453 spectrophotometer. The adsorption capacity at different time intervals was calculated using (Equation 1).

(1) $$\mathrm{qt}=\left(C_{0}-C_{t}\right)V/m$$

where C_t_ and C_0_ represent the MB concentration at time *t* and the initial concentration (mg⋅L^*–*1^), respectively. V is the volume of the reaction suspension, and m is the mass of the adsorbent [1]. To explore the adsorption isotherm, the initial MB concentration was varied between 5, 15, 30, 45 and 100 ppm. The adsorption capacity was modeled using Langmuir (Equation 2) and Freundlich (Equation 3) isotherms.

(2) $$\mathrm{qe}=\left(q_{m}K_{L}C_{e}\right)/\left(1+K_{L}C_{e}\right)$$

where q_e_ and q_m_ denote the equilibrium adsorption capacity (mg⋅g^*–*1^) and the monolayer adsorption capacity (mg⋅g^*–*1^), respectively. *K*_L_ is Langmuir constant (L⋅mg^*–*1^), and C_e_ is equilibrium concentration of MB [20].

(3) $$\mathrm{qe}=K_{F}Ce^{1/n}$$

where *K*_F_ and *n* are Freundlich constant and Freundlich exponent, respectively [20].

Thermodynamic parameters were obtained by measuring the adsorption capacity at 30, 50, and 70°C. The distribution coefficient *K*_c_ (defined as q_e_/C_e_) was used to fit the data to the van’t Hoff and Gibbs Equation:

(4) $$\ln Kc=\left(\Delta S/R\right)-\left(\Delta H/\mathrm{RT}\right)$$

(5) ΔG =ΔH −TΔS

where *G*, *H* and *S* are Gibbs free energy, enthalpy and entropy, respectively. R is gas constant (J⋅mol^*–*1^⋅K^*–*1^) and T is temperature [17].

### 2.5. Photocatalytic activity test

The photocatalytic performances of each sample (6 mg) were assessed through the degradation of a methylene blue (MB) aqueous solution (30 ppm, 300 mL) under visible light illumination. A LED-based visible light simulator (420–800 nm) with an intensity of 100 mW/cm^2^ was used as a light source. Prior to light exposure, the reaction solution was magnetically stirred in the dark for 30 min to ensure adsorption equilibrium. Samples were taken every hour, and the remaining MB concentration was measured using an Agilent HP 8453 spectrophotometer. For the reusability test, the CuO/ZnO-montmorillonite photocatalyst was recovered after each photocatalytic reaction by centrifugation. The solid was further washed several times with deionize water and ethanol to remove any adsorbed MB, then dried at 60°C before reused in subsequent photocatalytic cycles.

### 2.6. Characterization

Powder X-ray diffraction (XRD) patterns were recorded on a Bruker D8 ADVANCE diffractometer (USA) using monochromatic CuKα radiation (λ = 1.5406 Å). Scanning electron microscopic (SEM) images were taken on a SEC Mini-SEM SNE-4500 M scanning electron microscope (South Korea). Transmittance electron microscopic (TEM) images were taken on a Tecnai G^2^ 20 transmission electron microscope (China) operating at 200 kV. Diffuse reflectance absorption spectra of the powder products were recorded in the range of 200–1200 nm using a Shimadzu UV-VIS-NIR3101PC scanning spectrophotometer (Japan). Photoluminescence (PL) spectra of the solid samples were measured using a Shimadzu RF-5301PC spectrofluorophotometer (Japan) with the excitation wavelength of 350 nm. Nitrogen adsorption/desorption isotherms were obtained on a Micromeritics ASAP 2010 equipment (USA), after degassing at 300°C for 3 h.

## 3. Results and discussion

**Figure 1.**
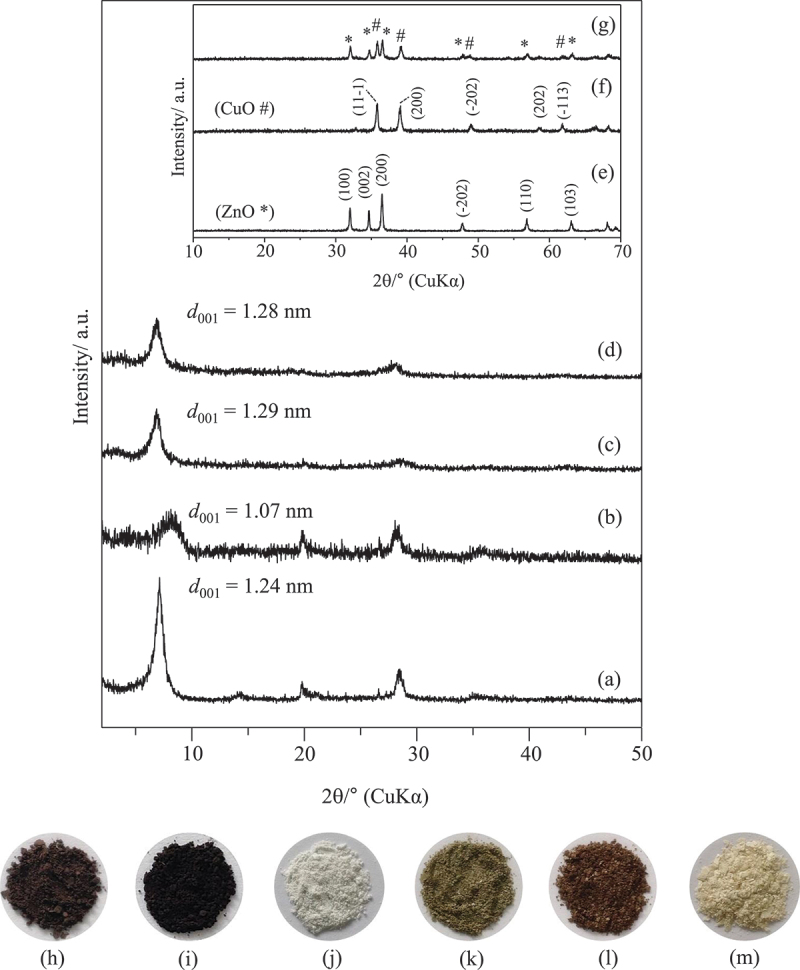
XRD patterns of (a) montmorillonite, (b) heated montmorillonite, (c) CuO/ZnO-montmorillonite, (d) heated CuO/ZnO-montmorillonite, (e) ZnO, (f) CuO, and (g) CuO/ZnO, and powder appearance of (h) CuO/ZnO, (i) CuO, (j) ZnO, (k) CuO/ZnO-montmorillonite (l), CuO-montmorillonite, and (m) ZnO-montmorillonite.

The XRD patterns of CuO/ZnO-montmorillonite as well as pristine montmorillonite, bare CuO/ZnO, bare CuO, and bare ZnO are shown in Figure 1. Following hydrothermal treatment, the basal spacing (*d*_001_) of CuO/ZnO-montmorillonite was increased to 1.29 nm (Figure 1(c)), compared to that of pristine montmorillonite (1.24 nm, Figure 1(a)). The larger *d*_001_ value might be caused by further hydration of the intercalated species. In order to investigate this, CuO/ZnO-montmorillonite and montmorillonite were heated at 200°C for 2 h. After heating, the *d*_001_ value of montmorillonite decreased from 1.24 nm to 1.07 nm, indicating the dehydration of the interlayer space (Figure 1(b)). The intensity of *001* diffraction also decreased, indicating the disordered alignment of the layers. Meanwhile, both the heated and as-prepared CuO/ZnO-montmorillonite samples showed the similar *d*_001_ values (1.28–1.29 nm) (Figure 1(d)), implying that the water molecules were not present in the interlayer space as with the pristine montmorillonite. The interlayer expansion in CuO/ZnO-montmorillonite was calculated as 0.32 nm, determined by subtracting the thickness of the silicate layer (0.96 nm) from the basal spacing of the heated CuO/ZnO-montmorillonite (1.28 nm). CuO/ZnO appeared dark brown (Figure 1(h)), while CuO/ZnO-montmorillonite exhibited greenish brown (Figure 1(k)). The color difference between CuO/ZnO-montmorillonite and CuO/ZnO is likely due to variation in the sizes and size distributions of the semiconductor particles. The interlayer expansion and color change in CuO and/or ZnO-montmorillonite suggested the formation of CuO and/or ZnO in the interlayer space or on the external surface of montmorillonite [12,21]. In addition, no reflection corresponding to CuO or ZnO (Figure 1(e,f)) [22] was observed in the XRD patterns of CuO/ZnO-montmorillonite (Figure 1(d)), CuO-montmorillonite (Figure S1b) and ZnO-montmorillonite (Figure S1d). These suggest that CuO and/or ZnO particles were primary formed as small nanoparticles within the interlayer space of montmorillonite; however, small particles on the external surface may also exist.

**Figure 2.**
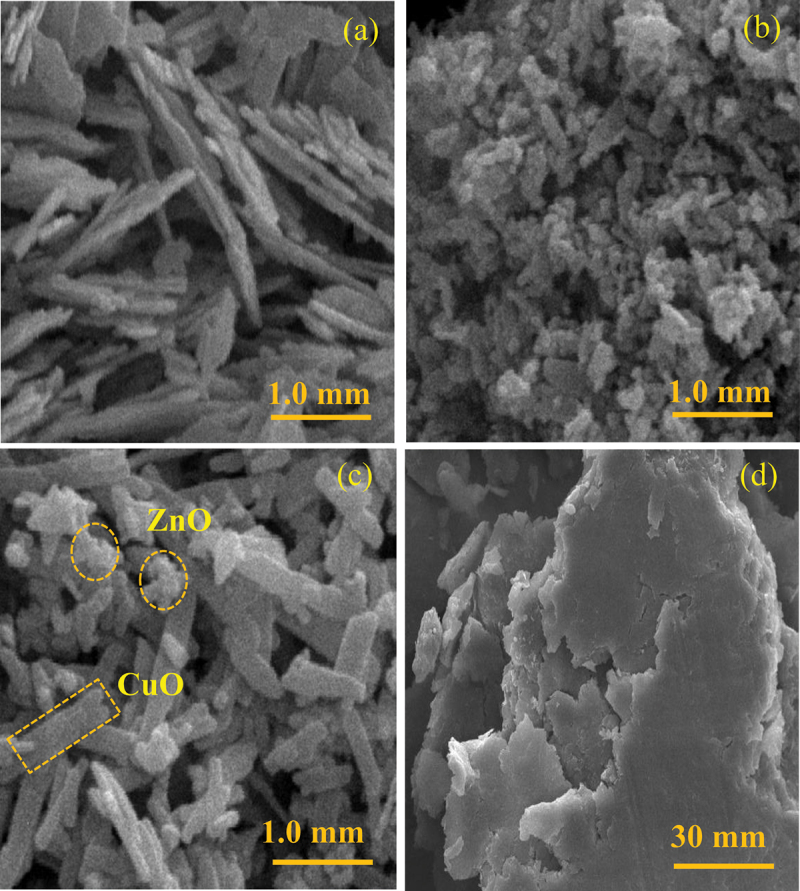
SEM micrographs of (a) CuO, (b) ZnO, (c) CuO/ZnO, and (d) CuO/ZnO-montmorillonite.

**Figure 3.**
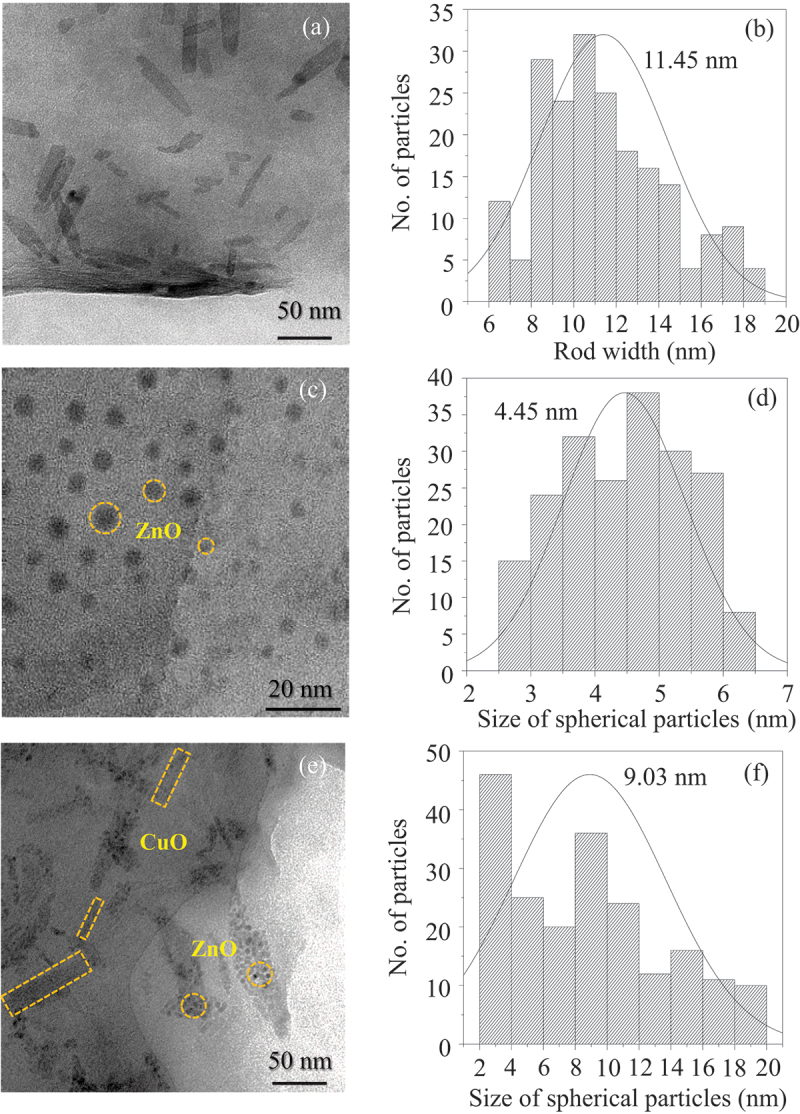
TEM micrographs and rod width/spherical particle size distribution of (a,b) CuO-montmorillonite, (c,d) ZnO-montmorillonite and (e,f) CuO/ZnO-montmorillonite. The rod widths of CuO were measured on their short sides of the rods.

SEM images reveal that CuO exhibited a rod-shaped morphology, while ZnO appeared irregularly shaped (Figure 2(a,b)). Both the rod-shaped CuO and irregularly shaped ZnO were observed in the SEM image of CuO/ZnO (Figure 2(c)), confirming the formation of CuO and ZnO particles. Meanwhile, CuO/ZnO-montmorillonite exhibited a sheet-like morphology characteristic of montmorillonite (Figure 2(d)). TEM images show that, in CuO-montmorillonite, CuO nanorods were formed and well distributed on the inside or surface of montmorillonite with an average rod width of 11.5 nm (Figure 3(a,b)). In contrast, ZnO-montmorillonite displayed homogeneously dispersed ZnO nanodots within the interlayer spaces of montmorillonite, with an average size of 4.5 nm (Figure 3(c,d)). These results suggest that the nanoenvironment provided by montmorillonite along with the reaction conditions, effectively prevented the aggregation of CuO and ZnO particles. TEM images of CuO/ZnO-montmorillonite (Figure 3(e)) revealed the presence of nanorods (in rectangular frame) and nanodots (in circle frame). The morphologies correspond to CuO and ZnO, confirming the successful incorporation of a CuO and ZnO mixture, which distributed uniformly in montmorillonite. The average size of spherical ZnO particle is 9.0 nm (Figure 3(f)). The sizes of spherical and rod-shaped particles, observed by SEM are much larger than this interlayer spacing. Considering the interlayer space between montmorillonite layers (approximately 0.2 nm), the expected height of deposited nanoparticles is very small. Given that the sizes of the spherical and rod-shaped particles are significantly larger than the interlayer spacing, it is expected that the montmorillonite layers undergo bending to accommodate and coat the deposited nanoparticles [23]. However, based on the XRD results related to dehydration discussed earlier, some ZnO or CuO clusters are likely present within the interlayer regions.

**Figure 4.**
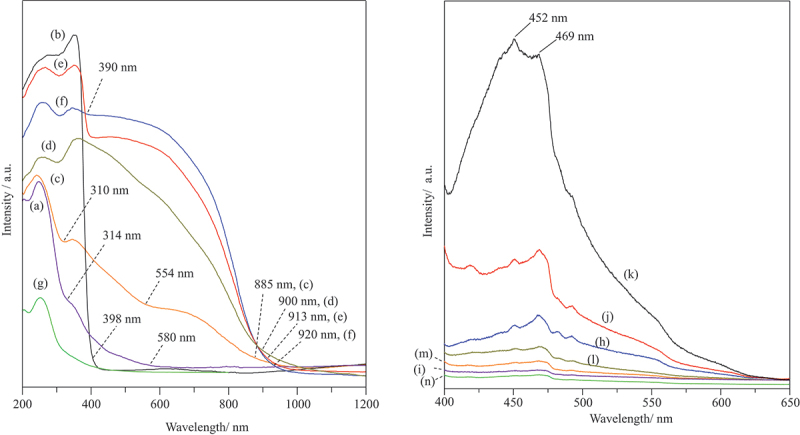
Diffuse reflectance absorption spectra of (a) ZnO-montmorillonite, (b) ZnO, (c) CuO/ZnO-montmorillonite, (d) CuO-montmorillonite, (e) CuO/ZnO, (f) CuO and (g) montmorillonite, and photoluminescence emission spectra (λ_ex_ = 350 nm) of (h) ZnO-montmorillonite, (i) CuO/ZnO-montmorillonite, (j) ZnO, (k) CuO/ZnO, (l) CuO-montmorillonite, (m) CuO, and (n) montmorillonite.

The data of the visible light-absorption onsets, band gap values, as well as luminescence bands and intensities of all samples are summarized in Table 1. The absorption band observed at approximately 250–260 nm in all the samples is attributed to the ligand to metal charge transfer (LMCT) from oxo species (O^2–^, OH^–^ or H_2_O) to ferric ions in montmorillonite [24]. The two absorption onsets at 314 and 580 nm were observed in the absorption spectra of ZnO-montmorillonite (Figure 4(a)), whereas only one absorption onset at 398 nm was observed for the bare ZnO (Figure 4(b)). The two absorptions are attributed to those of montmorillonite and ZnO, respectively. The longer absorption onsets of ZnO in ZnO-montmorillonite may be explained by the presence of defect due to nanoparticle morphology [25] and/or the Cu-doing in ZnO [26]. The CuO/ZnO-montmorillonite exhibited three absorption onsets at 885, 554, and 310 nm (Figure 4(c)), corresponding to the onsets observed in the spectra of CuO-montmorillonite (Figure 4(d)), ZnO-montmorillonite (Figure 4(a)), and montmorillonite (Figure 4(g)), respectively, with slight shifting. The blue-shift observed for CuO/ZnO-montmorillonite suggests the formation of smaller CuO and ZnO particles with montmorillonite [12,21,27]. The band gap energies of the metal oxides, calculated using Tauc plots [28] were 1.41 and 3.20 eV for CuO/ZnO (Figure S2c) as well as 1.48, 2.50, and 3.97 eV for CuO/ZnO-montmorillonite (Figure S2f). The presence of two distinct band gaps in the CuO/ZnO-montmorillonite composite, along with that of montmorillonite, confirms the coexistence of both CuO and ZnO phases, despite the possibility of minor Cu doping into ZnO or Zn doping into CuO. This band structure indicates that the photocatalyst is capable of activation under visible light irradiation, corresponding to energy gaps of 1.48 eV and 2.50 eV. The band edge positions of the ZnO and CuO were calculated using the following Equations [29]:

(6) $$E_{\mathrm{VB}}=\chi -E_{c}+1/2E_{g}$$

(7) $$E_{\mathrm{CB}}=E_{\mathrm{VB}}-E_{g}$$

**Table 1.** Basal spacing after heat treatment (200°C), absorption onset, band gap energy, and emission intensity (at 469 nm) of all products.

Sample	Basal spacing (nm)	Visible light-λ_onset_ (nm)	Visible light-band gap (eV)	PL intensity (a.u.) at 469 nm
CuO	–	920	1.44	–
ZnO	–	–	–	110
CuO/ZnO	–	913	1.41	58
CuO-montmorillonite	1.25	900	1.45	38
ZnO-montmorillonite	1.28	580	–	456
CuO/ZnO-montmorillonite	1.28	554, 885	1.48, 2.50	20

where the E_CB_ and E_VB_ represent the band edge positions of the conduction band and valence band, respectively, χ is the absolute electronegativity, which is 5.89 eV for ZnO and 5.81 eV for CuO, respectively [30,31], E_c_ refers to the energy of free electrons on the hydrogen scale (4.5 eV), and E_g_ is the band gap of the semiconductors. ZnO was calculated to have an estimated E_CB_ of −0.18 eV and E_VB_ of −2.96 eV, while CuO exhibited values of 0.59 eV and 2.03 eV, respectively. Furthermore, the flow of the charge carriers within the heterojunctions of CuO/ZnO-montmorillonite (Figure S3) might retard the recombination rate of the photo active species, thereby enhancing the photocatalytic efficiency.

Photoluminescence (PL) spectroscopy was conducted for understanding carrier recombination behaviors of the samples (Figure 4(h-n)). The bare ZnO exhibited PL emission bands in the range of 400–500 nm, attributed to intrinsic defects including oxygen vacancies, zinc vacancies and surface defects (Figure 4(j)) [32–34]. Meanwhile, CuO displayed a weak emission band in the same region (Figure 4(m)). The emission intensity of CuO/ZnO mixture in montmorillonite (Figure 4(i)) and ZnO in montmorillonite (Figure 4(h)) increased significantly, suggesting a quantum confinement effect due to the well-distributed semiconductor particles in montmorillonite [32–34]. Notably, the photoluminescence intensity of CuO/ZnO-montmorillonite was weaker than that of ZnO-montmorillonite (Table 1), indicating that the simultaneous presence of CuO and ZnO in montmorillonite may suppress the recombination rate of the photogenerated charge carriers, thus enhancing the photocatalytic activity.

**Figure 5.**
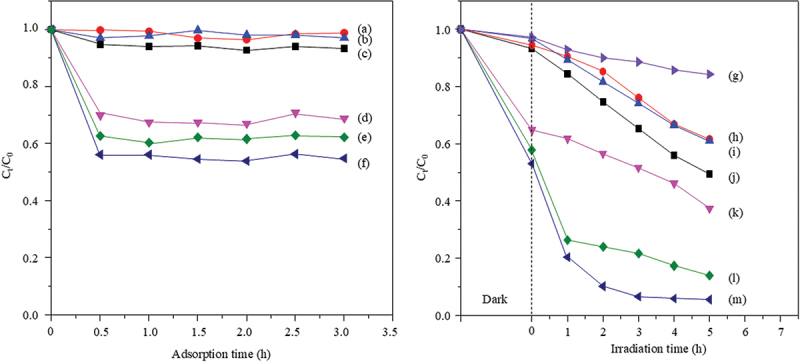
Adsorption activities of (a) ZnO, (b) CuO, (c) CuO/ZnO, (d) ZnO-montmorillonite, (e) CuO-montmorillonite, and (f) CuO-ZnO-montmorillonite in the dark, and photocatalytic behaviors of (g) MB photolysis, (h) ZnO, (i) CuO, (j) CuO/ZnO, (k) ZnO-montmorillonite, (l) CuO-montmorillonite, and (m) CuO/ZnO-montmorillonite under visible-light irradiation.

**Figure 6.**
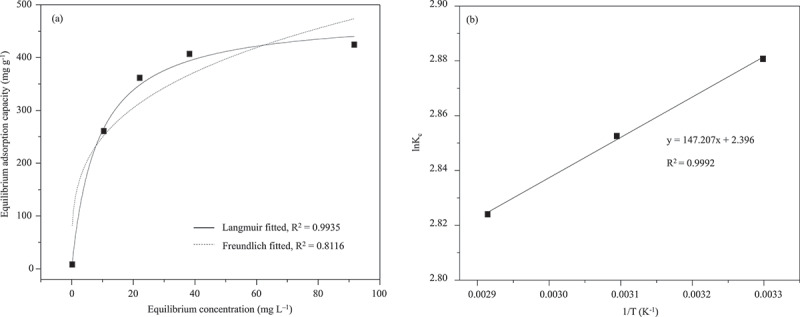
(a) Fitted adsorption isotherm and (b) relationship between lnK_c_-1/T of CuO/ZnO-montmorillonite adsorbent.

The adsorption capacities of MB for ZnO, CuO, CuO/ZnO, ZnO-montmorillonite, CuO-montmorillonite, and CuO/ZnO-montmorillonite were calculated to be 24, 36, 42, 297, 318 and 430 mg⋅g^−1^, respectively (Figure 5(a)). The adsorption kinetic of MB on all the adsorbents conformed more closely to the *pseudo*-second-order model than to the *pseudo*-first-order model. Among the tested materials, CuO/ZnO-montmorillonite exhibited the highest adsorption rate constant of 0.0296 g⋅mg^−1^⋅min^−1^ (Table 2). The drastic increase in the adsorption capacity and rate for the metal oxides was attributed to the increase in the specific surface area from 5 m^2^⋅g^−1^ for CuO/ZnO to 11 m^2^⋅g^−1^ for CuO/ZnO-montmorillonite after compositing with the montmorillonite. CuO/ZnO-montmorillonite, which revealed the highest adsorption capacity, was selected as a model to investigate the adsorption isotherm and thermodynamic. The adsorption data at various initial dye concentrations fit well with the Langmuir isotherm with a regression constant, R^2^ >0.99 (Figure 6(a)). The adsorbed MB formed a monolayer on the surface of CuO/ZnO-montmorillonite with the monolayer adsorption capacity of 454 mg⋅g^−1^. Thermodynamic parameters, including the change of Gibbs free energy (Δ*G*), enthalpy (Δ*H*), and entropy (Δ*S*), were calculated from the linearized van’t Hoff Equation (Figure 6(b)). The adsorption of MB on the CuO/ZnO-montmorillonite surface was spontaneous and exothermic, as indicated by the negative values of Δ*G* (−7.6 ± 0.4 kJ⋅mol^−1^) and Δ*H* (−1.2 kJ⋅mol^−1^). The small positive value of Δ*S* (0.02 kJ⋅K^−1^⋅mol^−1^) suggests an increase in the randomness of MB molecules on the adsorbent surface [20]. This phenomenon is likely due to the replacement of water molecules in the hydration shell of the adsorbent by the MB molecules [20]. The cationic MB molecules were adsorbed onto the anionic surface of montmorillonite (pH_pzc_ = 3.4 ± 0.2) [35] through electrostatic interactions.

**Table 2.** The *pseudo*-first order and *pseudo*-second order rate constants for adsorption and photocatalytic degradation of methylene blue.

Sample	Adsorption				Photodegradation			
	*pseudo*-first order (min^−1^)	R^2^	*pseudo*-second order (g⋅mg^−1^⋅min^−1^)	R^2^	*pseudo*-first order (min^−1^)	R^2^	*pseudo*-second order (g⋅mg^−1^⋅min^−1^)	R^2^
CuO	0.00005	0.2654	0.0162	0.9997	0.0017	0.9976	0.0064	0.9784
ZnO	0.00002	0.0286	0.0160	0.9998	0.0016	0.9988	0.0066	0.9902
CuO/ZnO	0.00008	0.3878	0.0163	0.9999	0.0023	0.9987	0.0060	0.9815
CuO-montmorillonite	0.00050	0.3125	0.0247	0.9985	0.0026	0.9941	0.0198	0.9563
ZnO-montmorillonite	0.00010	0.0867	0.0230	0.9988	0.0020	0.9910	0.0086	0.9654
CuO/ZnO-montmorillonite	0.00070	0.2466	0.0296	0.9973	0.0053	0.9997	0.0508	0.9842

The photocatalytic activity of the products was evaluated by the degradation of MB under visible light irradiation as shown in Figure 5(b). The photocatalytic activity of CuO/ZnO-montmorillonite increased continuously with the irradiation time, even in the absence of any added oxidants. CuO/ZnO-montmorillonite demonstrated a photocatalytic efficiency of 94% after 5 h, which was higher than that of CuO-montmorillonite (86%), ZnO-montmorillonite (63%), CuO/ZnO (51%), CuO (39%), and ZnO (38%). The photodegradation of methylene blue (MB) was better described by a pseudo-first-order kinetic model rather than a pseudo-second-order model, with CuO/ZnO-montmorillonite achieving the highest rate constant of 0.0053 min^−1^ (Table 2). The high photocatalytic activity and rate can be attributed to the coupling of CuO and ZnO in montmorillonite, which promoted charge carrier transport at the heterojunction interface, and inhibited the recombination of e^−^–h^+^ pairs. The effective charge carrier separation was confirmed by the weaker PL intensity of CuO/ZnO-montmorillonite (Figure 4(i)) compared to that of ZnO-montmorillonite (Figure 4(h)). Additionally, the enhanced photocatalytic activity may also result from the advantages of montmorillonite as a host material, which can: (1) control the particle size of semiconductor photocatalyst in the interlayer nanospace, or prevent the aggregation of the semiconductor photocatalyst, (2) act as an electron acceptor to delay the charge carrier recombination and (3) provide high adsorption capacity due to its large specific surface area [36].

**Figure 7.**
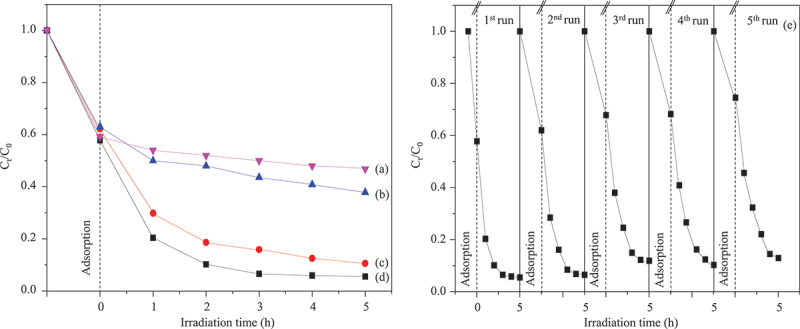
Photocatalytic behaviors of CuO/ZnO-montmorillonite with the presence of three scavengers (a) isopropyl alcohol, (b) EDTA-2Na^+^, (c) benzoquinone and (d) without scavenger, and (e) recycling test of CuO/ZnO-montmorillonite for 5 cycles run.

Three types of scavengers were individually added to the reaction solution to identify the major active species involved in the photocatalytic process. The photocatalytic efficiency of CuO/ZnO-montmorillonite decreased in the presence of benzoquinone, disodium ethylenediaminetetraacetate (EDTA-2Na^+^) and isopropyl alcohol, which serve as scavengers for superoxide radical (O_2_^∙–^), hole (h^+^), and hydroxyl radical (OH^∙^), respectively (Figure 7(a)) [37]. These results suggest that hydroxyl radicals (OH^∙^) were the most active species in the photocatalytic degradation of MB using CuO/ZnO-montmorillonite, followed by h^+^ and O_2_^∙–^, respectively. The mechanism of MB photocatalytic degradation was proposed based on the band potentials of ZnO and CuO semiconductors (Figure S3). Initially, MB dyes are adsorbed onto the surface of montmorillonite [2]. Then, most CuO particles and some larger-sized ZnO particles (λ_onset_ ≥390 nm) absorb the photons, generating charge carriers. Both ZnO and CuO reduces adsorbed oxygen to O_2_^∙–^, which subsequently decomposes MB into less toxic dye derivatives. Simultaneously, the excited electron in the conduction band of ZnO particles are transferred to the conduction band of CuO in hybrid, and some of them are also transferred to Fe impurity in the montmorillonite host [2]. The process inhibited the recombination of the exciton carriers, allowing the generation of abundant O_2_^∙–^ species to drive dye photodegradation [36]. Meanwhile, the holes in the valence band of ZnO are transferred to the valence band of CuO, contributing to hole delocalization and further inhibiting the e^–^ and h^+^ recombination, as evidenced by the weak activity of EDTA-2Na^+^ scavenger (Figure 7(b)) [36]. Adsorbed hydroxide ions are oxidized to hydroxyl radicals (OH^∙^) [37], which play a key role in attacking and degrading MB molecule. As shown in Figure S3, the h^+^ accumulates in the valence bands of both ZnO and CuO, without being transferred to any electron donor. This mechanism aligns with the primary pathway for the MB photodegradation *via* OH^∙^ species, consistent with the weak activity of isopropyl alcohol scavenger (Figure 7(a)). The removal efficiency of MB over CuO/ZnO-montmorillonite decreased by 5% (from 94 to 89%) after five cycle runs, likely due to the slight decrease in the adsorption capacity. Due to its high activity and stability, CuO/ZnO-montmorillonite has potential as an alternative photocatalyst for water pollution remediation under visible light irradiation, showing comparable photocatalytic activity to the previous studies (Table S1).

## 4. Conclusions

CuO/ZnO-montmorillonite was successfully synthesized through the reaction of an aqueous dispersion of Cu^2+^/Zn^2+^-montmorillonite with sodium hydroxide under hydrothermal reaction. The transmittance electron microscope, along with UV-visible and photoluminescence emission spectra, confirmed the formation of CuO nanorods and ZnO nanoparticles within the CuO/ZnO-montmorillonite structure. The CuO/ZnO-montmorillonite photocatalyst exhibited better degradation of methylene blue compared to single metal oxides embedded in montmorillonite. The enhanced performance is attributed to the efficient separation of photoexcited electrons and holes at the heterojunction interface, along with the increased specific surface area. The successful incorporation of CuO/ZnO in montmorillonite may inspire researchers to design novel low-dimensional nanohybrid materials as efficient visible-light driven photocatalysts for future innovation.

## Supplementary Material

Supplemental Material
