# Supplementary material for: Enhancement of photocatalytic efficiency of copper oxide/zinc oxide-montmorillonite photocatalyst under visible light irradiation
Source: Sci Technol Adv Mater. 2025 Feb 21;26(1):2469484. doi: 10.1080/14686996.2025.2469484 (PMC11905314; doi:10.1080/14686996.2025.2469484)
Supplement: Supplemental Material [file TSTA_A_2469484_SM7290.doc]

Supplementary information

**Enhancement of photocatalytic efficiency of copper oxide/zinc oxide-montmorillonite photocatalyst under visible light irradiation**

Chomponoot Suppaso^a^, Nipaporn Pongkan^a^, Sonchai Intachai^a,b^, Wachiraya Rattanawongsa^a^, Areebhorn Baoulan^c^, Yusuke Yamauchi^d,e^, Yusuke Asakura^d*^, Nithima Khaorapapong^a*^

*^a^ Materials Chemistry Research Center, Department of Chemistry and Center of Excellence for Innovation in Chemistry, Faculty of Science, Khon Kaen 40002, Thailand*

*^b^ Department of Chemistry, Faculty of Science, Thaksin University, Phatthalung, 93210, Thailand*

*^c^ Department of Chemistry, Faculty of Engineering, Rajamangala University of Technology Isan, Khon Kaen Campus, Khon Kaen 40000, Thailand.*

*^d^ Department of Materials Process Engineering, Graduate School of Engineering, Nagoya University, Nagoya, Aichi, Japan*

*^e^ School of Chemical Engineering, The University of Queensland (UQ), Queensland, Australia*

* Corresponding author

E-mail: [nithima@kku.ac.th](mailto:nithima@kku.ac.th), asa.y@nagoya-u.jp

**Table S1** Comparison of photocatalytic activities of various photocatalysts on methylene blue degradation under visible light irradiation with the present work

| Photocatalyst | % Dye removal | Kinetic constant (min^−1^) | Photocatalytic condition | | | | | Reference |
| --- | --- | --- | --- | --- | --- | --- | --- | --- |
|  |  |  | Concentration of MB (ppm) | Volume of MB (mL) | Amount of catalyst (mg) | Irradiation time/ lamp source | Other |  |
| CuO/ZnO-montmorillonite | 94 | 0.0053 | 30 | 300 | 6 | 300 min / 100 mW per cm^2^ LED-based visible light simulator  (λ = 420-800 nm) | pH = 7 ± 0.5 | This work |
| CuO-montmorillonite | 86 | 0.0026 |  |  |  |  |  |  |
| ZnO-montmorillonite | 63 | 0.0020 |  |  |  |  |  |  |
| CuO/ZnO | 51 | 0.0023 |  |  |  |  |  |  |
| CuO | 39 | 0.0017 |  |  |  |  |  |  |
| ZnO | 38 | 0.0016 |  |  |  |  |  |  |
| CuO- montmorillonite | 60.0 | - | 3.2 | 50 | 50 | 20 min/  29 mW per cm^−2^ tungsten lamp | pH = 8 | [1] |
| Montmorillonite | 10.0 | - |  |  |  |  |  |  |
| CuO | 24.3 | - |  |  |  |  |  |  |
| ZnO/CuO (95:5 %wt ratio) | 97.2 | - | ~ 9.6  (3×10^−5^ M) | 500 | 500 | 120 min/  250 W, 532 nm |  | [2] |
| ZnO/CuO (50:50 %wt ratio) | 38.5 | - |  |  |  |  |  |  |
| ZnO | 0.9 | - |  |  |  |  |  |  |
| Ag/ZnO-Mt | 82.5 | - | 3.2 | 100 | 7 | 50 min/100 w tungsten lamp (λ > 400 nm) | pH = 7 | [3] |
| ZnO-Mt | 15.7 | - |  |  |  |  |  |  |
| Ag/ZnO | 37.57 | - |  |  |  |  |  |  |
| Montmorillonite | 6.43 | - |  |  |  |  |  |  |
| ZnO-rectorite | 99.9 | - | 15 | 100 | 90 | 120 min/ 300W tungsten lamp |  | [4] |
| CuO/ZnO (20/80 %wt ratio) | 97 | - | 10 | 10 | 40 | 85 min/ 500 W tungsten halogen lamp | With 3.0 mL of H_2_O_2_ (0.08 M) | [5] |
| CuO/ZnO-g-C_3_N_4_ | 99 | 0.088 | ~ 3.2  (1×10^−5^ M) | 50 | 50 | 75 min/ 100 W Xe lamp | With 0.1 mmol of H_2_O_2_ | [6] |
| CuO/ZnO nanoparticle | 98.07 | - | 10 | 50 | 100 | 150 min/ Halogen lamp of 500 W |  | [7] |
| ZnO/CuO composite | 100 | 0.0532 | 100 | 500 | 400 | 90 min/ 160 W metal halide lamp (cut-off filter : λ ≤ 420nm) |  | [8] |
| CuO-PCH | 59 | 0.0215 | 10 | 100 | 8 | 60 min/ 200 W tungsten lamp  (λ > 400 nm) | pH = 9 | [9] |
| CuO-MCM-41 | 15 | 0.0088 |  |  |  |  |  |  |


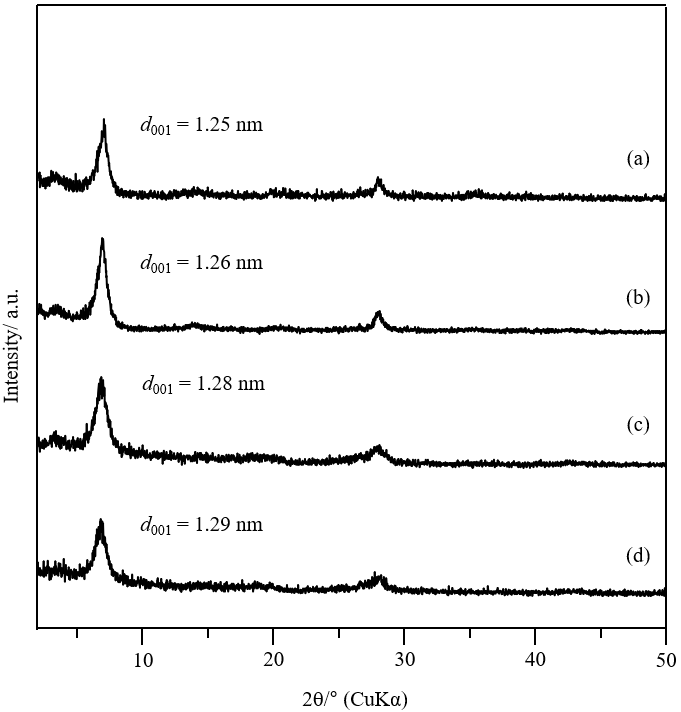


Figure S1. XRD patterns of (a) heated CuO-montmorillonite, (b) CuO-montmorillonite, (c) heated ZnO-montmorillonite, (d) ZnO-montmorillonite.


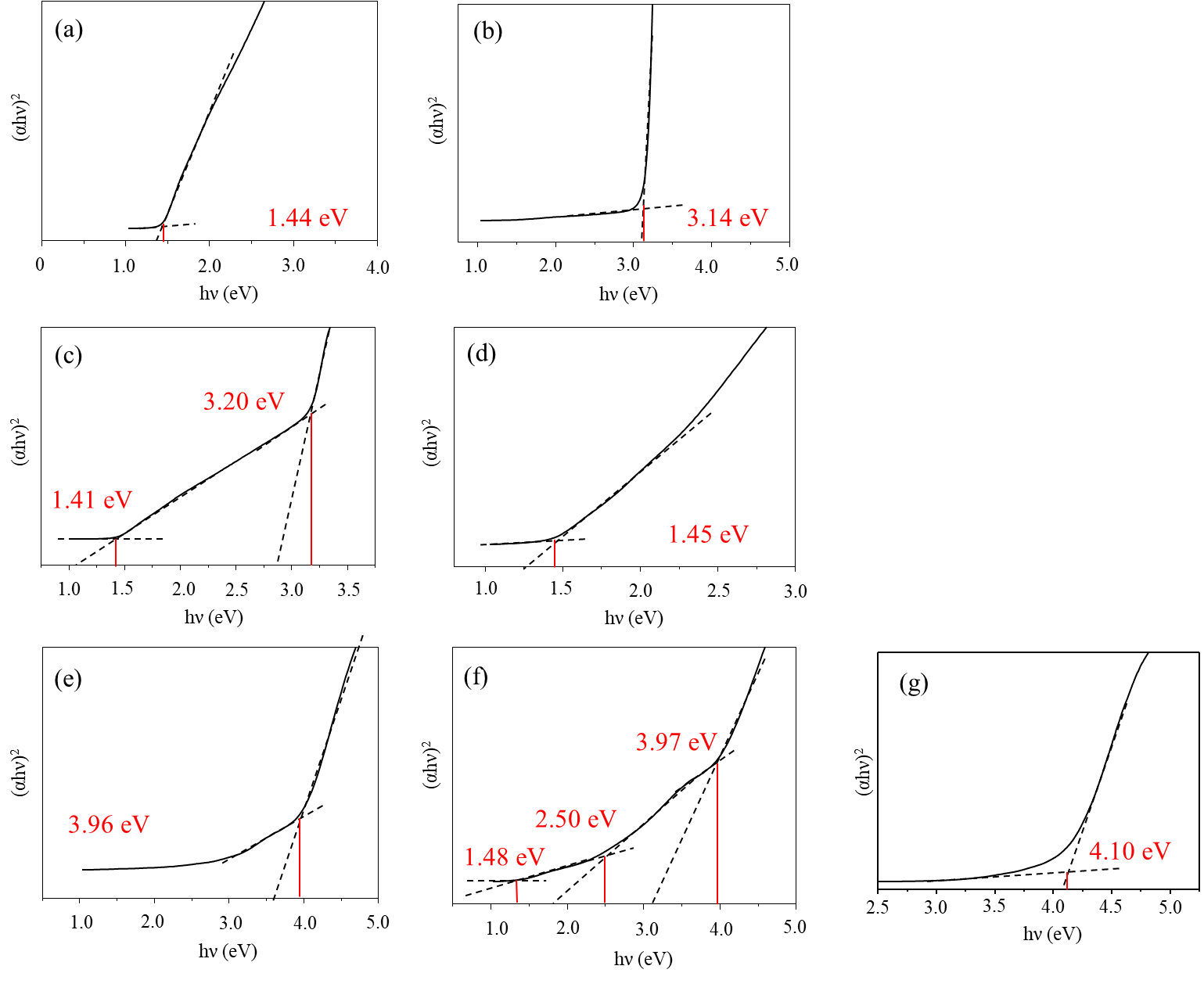


**Figure S2.** Tauc plot-band gap energy of (a) CuO, (b) ZnO, (c) CuO/ZnO, (d) CuO-montmorillonite, (e) ZnO-montmorillonite, (f) CuO/ZnO-montmorillonite, and (g) bare-montmorillonite


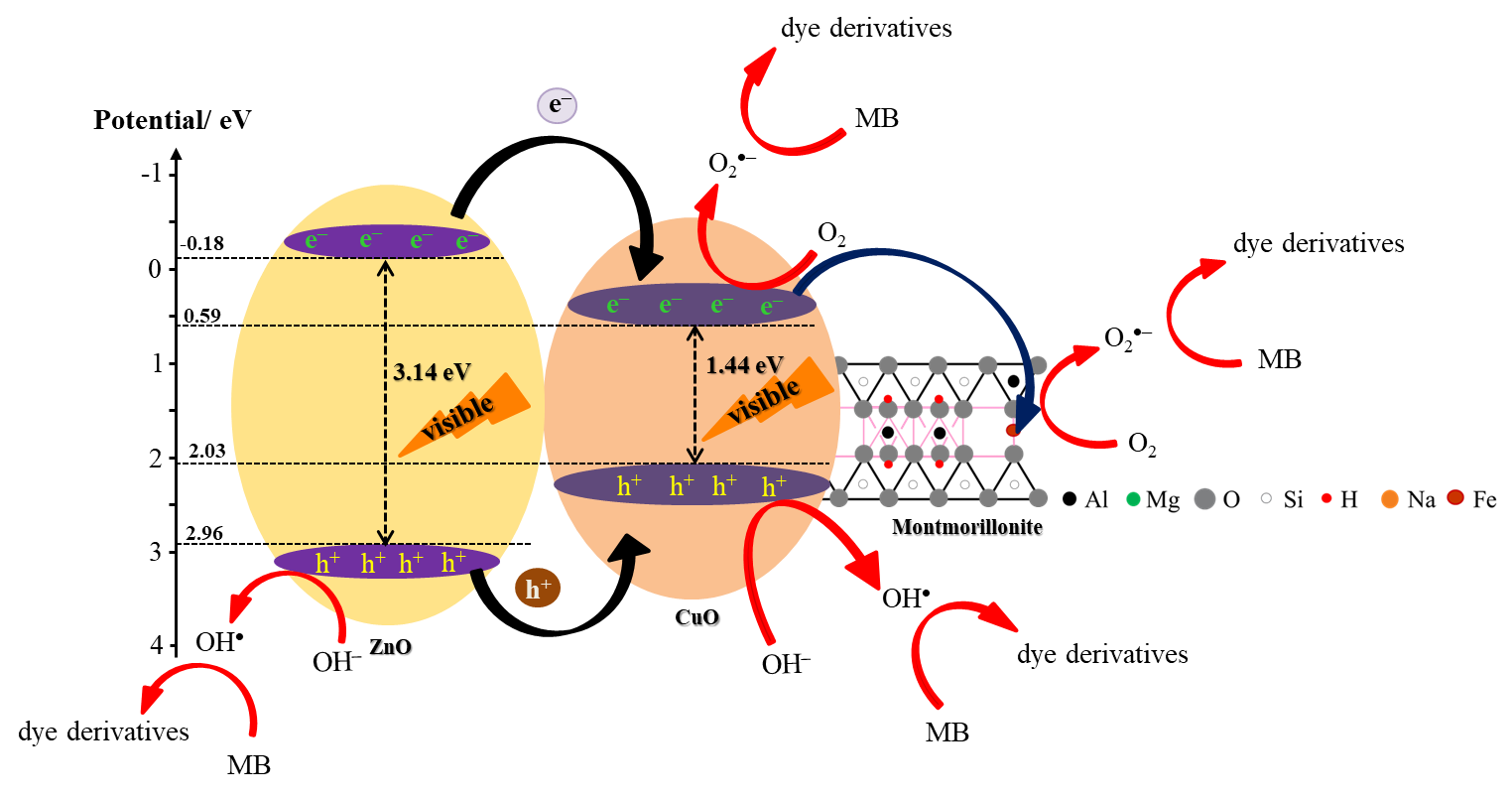


**Figure S3.** Revolution of the dye degradation of dye by CuO/ZnO and CuO/ZnO-montmorillonite under visible irradiation

**References**

[1] Sohrabnezhad S, Pourahmad A, Salavatiyan T. CuO–MMT nanocomposite: effective photocatalyst for the discoloration of methylene blue in the absence of H2O2. Applied Physics A. 2016 2016/02/01;122(2):111.

[2] Saravanan R, Karthikeyan S, Gupta VK, et al. Enhanced photocatalytic activity of ZnO/CuO nanocomposite for the degradation of textile dye on visible light illumination. Materials Science and Engineering: C. 2013 2013/01/01/;33(1):91-98.

[3] Sohrabnezhad S, Seifi A. The green synthesis of Ag/ZnO in montmorillonite with enhanced photocatalytic activity. Applied Surface Science. 2016 2016/11/15/;386:33-40.

[4] Li S-q, Zhou P-j, Zhang W-s, et al. Effective photocatalytic decolorization of methylene blue utilizing ZnO/rectorite nanocomposite under simulated solar irradiation. Journal of Alloys and Compounds. 2014 2014/12/15/;616:227-234.

[5] Fouda A, Salem SS, Wassel AR, et al. Optimization of green biosynthesized visible light active CuO/ZnO nano-photocatalysts for the degradation of organic methylene blue dye. Heliyon. 2020 2020/09/01/;6(9):e04896.

[6] Sivasakthi S, Gurunathan K. Graphitic carbon nitride bedecked with CuO/ZnO hetero-interface microflower towards high photocatalytic performance. Renewable Energy. 2020 2020/10/01/;159:786-800.

[7] AlSalhi MS, Sakthisabarimoorthi A, Devanesan S, et al. Study on photocatalytic and impedance spectroscopy investigations of composite CuO/ZnO nanoparticles. Journal of Materials Science: Materials in Electronics. 2019 2019/07/01;30(14):13708-13718.

[8] Minh TT, Tu NTT, Van Thi TT, et al. Synthesis of Porous Octahedral ZnO/CuO Composites from Zn/Cu‐Based MOF‐199 and Their Applications in Visible‐Light‐Driven Photocatalytic Degradation of Dyes. Journal of Nanomaterials. 2019;2019(1):5198045.

[9] Sohrabnezhad S, Takas ME. Synthesis and characterization of porous clay heterostructure intercalated with CuO nanoparticles as a visible light-driven photocatalyst. Journal of the Iranian Chemical Society. 2019 2019/01/01;16(1):45-55.
